# Topical use and systemic action of green and roasted coffee oils and ground oils in a cutaneous incision model in rats (*Rattus norvegicus albinus*)

**DOI:** 10.1371/journal.pone.0188779

**Published:** 2017-12-13

**Authors:** Bruno Grosselli Lania, Joseane Morari, Aglécio Luis de Souza, Marilene Neves da Silva, Amanda Roberta de Almeida, Gislaine Veira-Damiani, Sarah Monte Alegre, Carlos Lenz César, Lício Augusto Velloso, Maria Letícia Cintra, Nilson Borlina Maia, Paulo Eduardo Neves Ferreira Velho

**Affiliations:** 1 Laboratory of Applied Research in Dermatology and Bartonella Infection,–School of Medical Sciences–University of Campinas. Campinas, Sao Paulo, Brazil; 2 Cell Signaling Laboratory and Obesity and Comorbidities Research Center—School of Medical Sciences–University of Campinas. Campinas, Sao Paulo, Brazil; 3 Metabolism Laboratory–Internal Medicine—School of Medical Sciences–University of Campinas. Campinas, Sao Paulo, Brazil; 4 Clinical Medicine Department–School of Medical Sciences–University of Campinas. Campinas, Sao Paulo, Brazil; 5 National Institute of Photonics Applied to Cell Biology (INFABIC)–Gleb Wataghin Institute of Physics–University of Campinas. Campinas, Sao Paulo, Brazil; 6 Pathological Anatomy Department—School of Medical Sciences–University of Campinas. Campinas, Sao Paulo, Brazil; 7 Aromatic Plants Division—Agronomic Institute of Campinas. Campinas, Sao Paulo, Brazil; Ohio State University, UNITED STATES

## Abstract

**Introduction:**

Wounds are a common health problem. Coffee is widely consumed and its oil contains essential fatty acids. We evaluated the local (skin) and systemic effects associated with the topical use of coffee oils in rats.

**Methods:**

Punch skin wounds (6 mm) incisions were generated on the backs of 75 rats. Saline (SS), mineral oil (MO), green coffee oil (GCO), roasted coffee oil (RCO), green coffee ground oil (GCGO) or roasted coffee ground oil (RCGO) were topically applied to the wounds. Healing was evaluated by visual and histological/morphometric optical microscopy examination; second harmonics generation (SHG) microscopy, wound tissue q-PCR (values in fold-change) and blood serum (ELISA, values in pg/mL).

**Results:**

RCO treated animals presented faster wound healing (0.986 vs. 0.422), higher mRNA expression of IGF-1 (2.78 vs. 1.00, p = 0.01), IL-6 (10.72 vs. 1.00, p = 0.001) and IL-23 (4.10 vs. 1.2, p = 0.05) in early stages of wound healing; higher IL-12 (3.32 vs. 1.00, p = 0.05) in the later stages; and lower serum levels of IFN-γ (11.97 vs. 196.45, p = 0.01). GCO treatment led to higher mRNA expression of IL-6 (day 2: 7.94 vs. 1.00, p = 0.001 and day 4: 6.90 vs. 1.00, p = 0.01) and IL-23 (7.93 vs. 1.20, p = 0.001) in the early stages. The RCO treatment also produced higher serum IFN-α levels throughout the experiment (day 2: 52.53 vs. 21.20; day 4: 46.98 vs.21.56; day 10: 83.61 vs. 25.69, p = 0.05) and lower levels of IL-4 (day 4: 0.9 vs.13.36, p = 0.01), adiponectin (day 10: 8,367.47 vs. 16,526.38, p = 0.001) and IFN-γ (day 4: 43.03 vs.196.45, p = 0.05). The SHG analysis showed a higher collagen density in the RCO and GCO treatments (p = 0.05).

**Conclusion:**

Topical treatment with coffee oils led to systemic actions and faster wound healing in rats. Further studies should be performed are necessary to assess the safety of topical vegetal oil use for skin lesions.

## Introduction

Acute wounds are a common health problem that affects 11 million people and results in hospitalization of approximately 300,000 people hospitalized in the United States [1]. Aging, obesity, diabetes, cardiovascular disorders, sensory neuropathies, and autoimmune diseases delay wound healing. In 2009, chronic wounds affected 6.5 million people and had an annual cost of approximately $25 billion dollars in the United States[2].

Typically, acute wound healing is a well-organized process that lead to predictable tissue repair, with platelets, keratinocytes, immune surveillance cells, microvascular cells, and fibroblasts playing key roles in the restoration of tissue integrity [3]. This processes can be didactically divided into four overlapping stages: homeostasis, inflammation, proliferation and remodeling. Studies have shown that during this process, several pro-, and anti-inflammatory factors are produced either locally or systemically, including leptin [4], interleukin (IL)2, IL-4, IL-6, and insulin-related growth factor (IGF)-1 as pro-healing substances (important during initial healing stages); adiponectin, IL-12, interferon (IFN)-α, and IFN-γ [5] as anti-healing factors (important at later healing stages); and tumor necrosis factor (TNF)-α, which has a varying effect depending on its concentration [6].

For years, plants have been used in traditional medicine to treat a wide range of human diseases [7]. Nevertheless, biomaterials for tissue engineering are extremely expensive, and they require heavy expenses beyond the reach of most patients in developing countries. Therefore, medicinal plants could be useful for primary health care because they are inexpensive, safe, and effective natural products compared with synthetic drugs [8]. Although effective wound dressing may not be available, treatments that incorporate fresh homemade coffee powder have long been recognized as a traditional medicinal practice [9]. In addition, the use of green coffee oil in the cosmetic and pharmaceutical industries is increasing because of its composition, hydration properties and ability to improve the skin barrier [10].

Coffee is one the most consumed plants in the world and Brazil is the largest coffee producer and exporter, with an estimated production of more than 56 thousand 60-kg bags only in the 2016–2017 crop [11]. According to a study conducted by Oliveira and colleagues, approximately 1/5 of the total production results in defective beans, that are not suitable for beverage-use [12]. As an alternative, these beans could be used for medicinal and cosmetic applications [13].

The aim of this study is to evaluate the local and systemic actions of different types of topically–applied coffee oils.

## Materials and methods

Seventy five female Sprague-Dawley rats (strain NTacUnib:SD), with an median age of 7-week were obtained from the University of Campinas Breeding Center (*CEMIB*). The study was approved by the University of Campinas Institutional Animal Care and Use Committee (*CEUA* protocol number 2929–1) and was performed in accordance with the Brazilian College for Animal Experimentation and the International Council for Laboratory Animal Science (*ICLAS*) guidelines.

The sample size (15 animals per group, five for each analysis point) was pre-determined by the CEUA, which limits the number of animals in each group.

The animals were housed in individual polypropylene cages under the following conditions: 22°C, 12:12-hour artificial light-dark cycle and *ad libitum* access to food and drinking water. After random selection, the rats were anesthetized (ketamine 50 mg/kg-1, xylazine 7.0 mg/kg-1, and diazepam 2.0 mg/kg-1). Using 6.0 mm punches [14, 15], we produced four wounds on the left side and four on the right side of the back of each animal. The animals were randomly divided into 5 groups of 15 animals as follows: Group 1 –the lesions were only cleaned with saline solution (SS) on the left side and treated with mineral oil (MO) on the right side; Group 2 –the lesions were treated with MO on the left side and green coffee oil (GCO) on the right side; Group 3 –the lesions were treated with MO on the left side and roasted coffee oil (RCO) on the right side; Group 4 –the lesions were treated with MO on the left side and green coffee grounds oil (GCGO) on the right side; Group 5 –the lesions were treated with MO on the left side and roasted coffee grounds oil (RCGO) on the right side. Immediately after the wounds were produced, the first product application was performed on each lesion. The animals in Groups 2, 3, 4 and 5 were treated with topical MO on the left side wounds, the same considered as treatment on Group 1, to assess whether the coffee oils had a systemic effect on the animal. The lesions on each side were covered with different gauzes reinforced with tape to avoid admixtures of the different oils applied to the same animal. The animals had access to water with paracetamol to help control the pain. The wounds were cleaned with SS, the topical application of oils was repeated, and the bandages were replaced daily until the established euthanasia dates.

### Sample collection

After random selection for euthanasia on days 2, 4, or 10 (five animals each day), the rats were anesthetized with thiopental (85.0 mg/kg-1). Photographs were taken, and blood was immediately collected to measure healing-related substances using ELISA. Skin sections of the shaved dorsal areas of two wounds on each side were excised to the fascia, with the proximal halves separated, for histological analysis.

### Clinical evaluation

During the daily handling of each animal, the rate of wound contraction, the reduction of the erythema and edema, and the re-epithelization were visually assessed by comparing the lesions with each other and with previously taken photos.

### Wound area

Wounds were photographed on days two, four and 10 after surgical intervention with a constant focal length. The unhealed areas were quantified by the “number of pixels". The reduction relative to the topographic opposite (on the left side) was determined for each wound treated (on the right side). In this way, the proportion of the treated wound that remained unhealed after the experiment began could be quantified in relation to the control side of the same animal. This proportion was considered 100% on day zero [16].

### Histology

Sections of the euthanized animals were stained with hematoxylin and eosin (H&E) were blindly evaluated for the qualitative analysis, we evaluated the type of cells that made up the inflammatory environment. All animals presented fibroblasts and endothelial cells as well as few lymphomononuclear cells amid collagen bundles (mature granulation tissue). For the morphometric analysis (carried out in ten x 400 random fields and aided by an eyepiece with cycloids grid coupled to the ocular lens), all nuclei abutting the cycloids were counted on sections of the euthanized animals obtained on day 10 and the results were recorded as the arithmetical average, as previously described [17].

### SHG analysis

Nonlinear imaging was used to evaluate the collagen organization of the skin via the second harmonic generation (SHG) technique. Images from wound areas were acquired from the National Institute of Photonics Applied to Cell Biology (INFABIC) using a microscope (Zeiss Axio Observer.Z1 LSM780; Carl Zeiss, Oberkochen, Germany) with 512 × 512 pixels spatial resolution each. The sample was excited by a MaiTai Ti:Sapphire laser (Spectra Physics, Santa Clara, CA, USA) at a frequency of 800 nm and a 10x objective. For quantitative image analyses, we used the optical density tool in ImageJ (NIH, available from http://rsb.info.nih.gov/ij). Inside the wound, we selected the central area of the wound as the specific region of interest (ROI), and this box was used for all images.

### RNA extraction

Samples of the healed area of the skin were obtained using 6 mm punches in the same location of the original lesions to avoid collecting normal skin, with samples collected from the two lesions on the control side and two lesions on the treated side closest to the animal’s tail.

The wounds were homogenized in 1 mL of TRIzol reagent (Invitrogen, Carlsbad, CA, USA), and the total RNA was obtained following the manufacturer's instructions. A High Capacity cDNA Reverse Transcription Kit (Life Technologies, Carlsbad, CA, USA) was used to reverse transcribed three micrograms of total mRNA.

### Real-time PCR

For the PCR assay, each well contained 40 ng of cDNA, 0.5μl of the specific primer, 5.0 μl of TaqMan Universal Master Mix (4369016, Life Technologies, Carlsbad, CA, USA) and RNase -free water to a 10 μl final volume according to the manufacturer’s recommendation. TaqMan PCR Master Mix Gene expression analyses were performed on a Step One Plus™ Real-Time PCR System (Applied Biosystems, Foster City, CA, USA). Rat Gapdh was used as the reference gene for all samples. The reference gene and the specific primers were purchased from Applied Biosystems and consisted of the following: adiponectin (Rn00595250_m1)IL-2 (Rn00587673_m1), IL-4 (Rn01456866_m1), IL-6 (Rn01410330_m1), IL-12 (Rn00575112_m1), IL-23 (Rn00590334_g1), IFN-α (Rn01400027_g1), IFN-γ **(**Rn00594078_m1) and IGF-1(Rn00710306_m1),. The average of the control group (MO) on each treatment day (day 2, 4 or 10) was used as the calibrator sample for all groups. The PCR efficiency was calculated before the start of the experiments, and the 2^-DDCT method was used to calculate gene expression [18].

### ELISA

Serum was obtained by centrifuging of whole blood samples (10 min, 4°C, 3.500 RPM). This trial used the Quantitative Sandwich Enzyme Immunoassay Technique. Quantikine ELISA kits for IL-4, IL-12, IL-6, IGF-1, IFN-α and IFN-γ, were purchased from R&D Systems Minneapolis, MN, USA, and for leptin and adiponectin were purchased from Millipore, St. Charles. All plates were analyzed using a Bio-Rad 680 microplate reader, (Bio-Rad Laboratories, Hercules, CA, USA), at 450 nm and 570 nm wavelengths according to the manufacturer’s recommendations.

### Coffee oils extraction

The coffee oil was purchased wholesale from a trader of raw coffee beans produced by farmers in the region of Varginha, MG, Brazil.

Half of the beans were roasted in a Lilla -Coffee Roaster with capacity of 6 bags (60 kg/bag) of coffee beans (Lilla Inc, Guarulhos, SP, Brazil) following the typical method of producing coffee for domestic consumption in Brazil, and the beans were then processed at the cold -expeller press to extract the roasted coffee oil.

The second half was directly processed in the same press expeller. A Piratininga coffee -press adapted to extract coffee oil was used (Maquinas Piratininga Jaboatao dos Guararapes, PE, Brazil). Both oils were filtered in the filter press equipment. The green and roasted coffee grounds kept by the filter were used in the GCGO and RCGO treatments in this study.

### Coffee oils composition

The essential fatty acids composition of the green and roasted coffee oils were analyzed by gas chromatography following the methodology described by the American Oil Chemists’ Society (AOCS) [19]. The analysis conditions were as follows: capillary gas chromatograph (CGC), AGILENT 6850 SERIES GC SYSTEM (Agilent, Santa Clara, CA, USA); and capillary column, DB-23 AGILENT, with a (50% cyanopropyl)—methylpolysiloxane phase, 60 m length, 0.25 mm Ø int and 0.25 μm film. The operating conditions of the chromatograph were as follows: column flow rate = 1.00 mL/min; linear velocity = 24 cm/s; detector temperature = 280°C; injector temperature = 250°C; oven temperature = 110°C for 5 min, 110°C—215°C at 5°C/min, and 215°C for 34 min; carrier gas = helium; and volume injected = 1.0 μL.

The phenolic composition of the oils was analyzed via gas chromatography–mass spectrometry (GC-MS). Aliquots of 100 μL of the oil samples were derivatized with 500 μL of N,O-Bis(trimethylsilyl) trifluoroacetamide, Trimethylchlorosilane (BSTFA + TCMS) solution (99: 1) (Supelco, Bellefonte, PA, USA) at 60°C for 3 h. The dilution of derivatized sample was 1:10. The column set consisted of aTR-5 at 7 m × 32 mm (d_f_ = 0.25 μm). The volume injected was 1 μL using a split/splitless injector (1:50) with a closed purge for 3.0 min. The injection port was kept at 280°C, and the MS transfer lines were kept at 300°C. For all runs, the oven temperature programming was set to increase from 60°C to 240°C at 3°C min^-1^. The MS ionization source was maintained at 250°C and 70 eV, and the scan range was set from m/z 40–600 units (scanning interval, 0.064 s). Peak identification was performed by matching against the NIST 2010 (NIST–Gaithersburg, MD, USA) and the FFNSC (Chromaleont–Messina, Italy) spectra libraries [20, 21].

### Statistical analysis

Two-way analyses of variance (2-way ANOVAs) and Bonferroni post-tests were used at a significance level of 5% using GraphPad Prism software.

## Results

### Clinical evaluation

**Fig 1:**
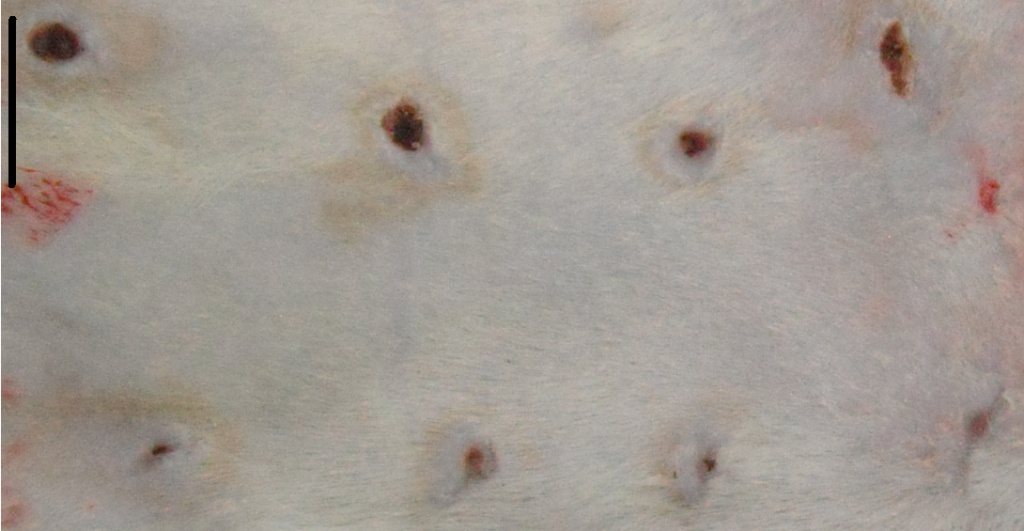
Representative image of the wounds on a control group animal. The wounds on the left were only cleansed daily with saline whereas the wounds on the right were treated with MO. This photograph was taken 10 days after the wounding procedure. Bar = 1 cm.

**Fig 2:**
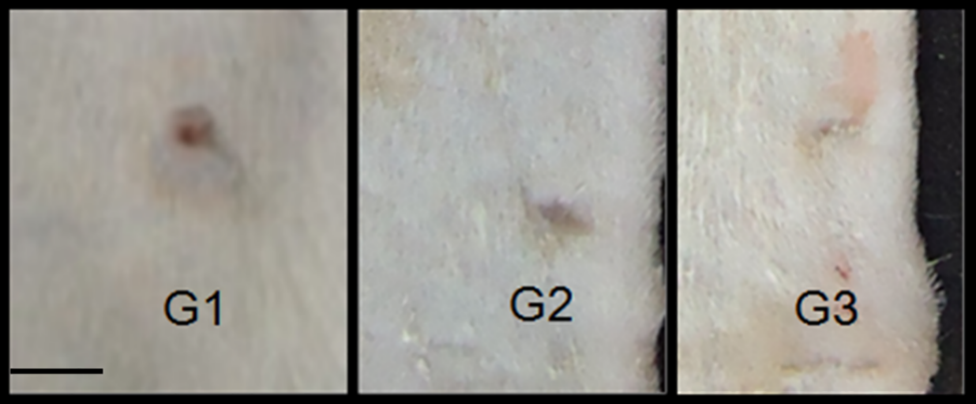
Representative image of wound treated with MO (G1), GCO (G2) and RCO (G3). Wound healing was faster in G3, although significant differences were not observed among the groups.

The visual evaluation performed during the daily manipulation of each animal (for performing treatment renewal, taking photographs and changing dressing) indicated that if the wound was kept moist for a longer period, the wounds healed better [22], which was noticeable in the control group animals as shown in Fig 1. The wounds on the right side, which received MO, show more advanced healing than the wounds on the left side, which were only cleansed with saline. A significant improvement in the Group 3 animals treated with RCO was noticeable compared with that of the other groups. In this group, more effective wound healing was observed starting on the second and third days. The GCGO and RCGO treatments induced the formation of crusts at the wound site, which led to worsened cicatrization. Therefore, these groups could not be analyzed. Fig 2 depicts representative images of Groups 1–5.

### Wound area

The ulcer areas measurement results (shown in Table 1) show that the wounds treated with RCO presented a higher healing speed (b) of the corresponding derivative. The second-degree derivative of the curves displays the effect, showing a two-fold faster healing process for the RCO treatment compared with the control group. The scarring area, however, was occasionally difficult to measure because of the presence of crusts that were not removed with the delicate cleansing. The crusts that persisted were not removed; therefore, the histological evaluations were the most relevant parameters.

**Table 1:** Wound area.

Treatment	Area Healed		Polynomial Regression Coefficients		Healing speed *
	25%	50%	a	b	- δ “
G1 MO	4.72	7.7	-5.9	0.422	0.422
G2 GCO	4.9	8.5	-2.8	0.622	0.622
G3 RCO	3.9	6.7	-3.7	0.986	0.986

Wound area healed, polynomial regression coefficients and healing speed of Groups 1–3.

* Second-degree derivative

### Histology

**Fig 3:**
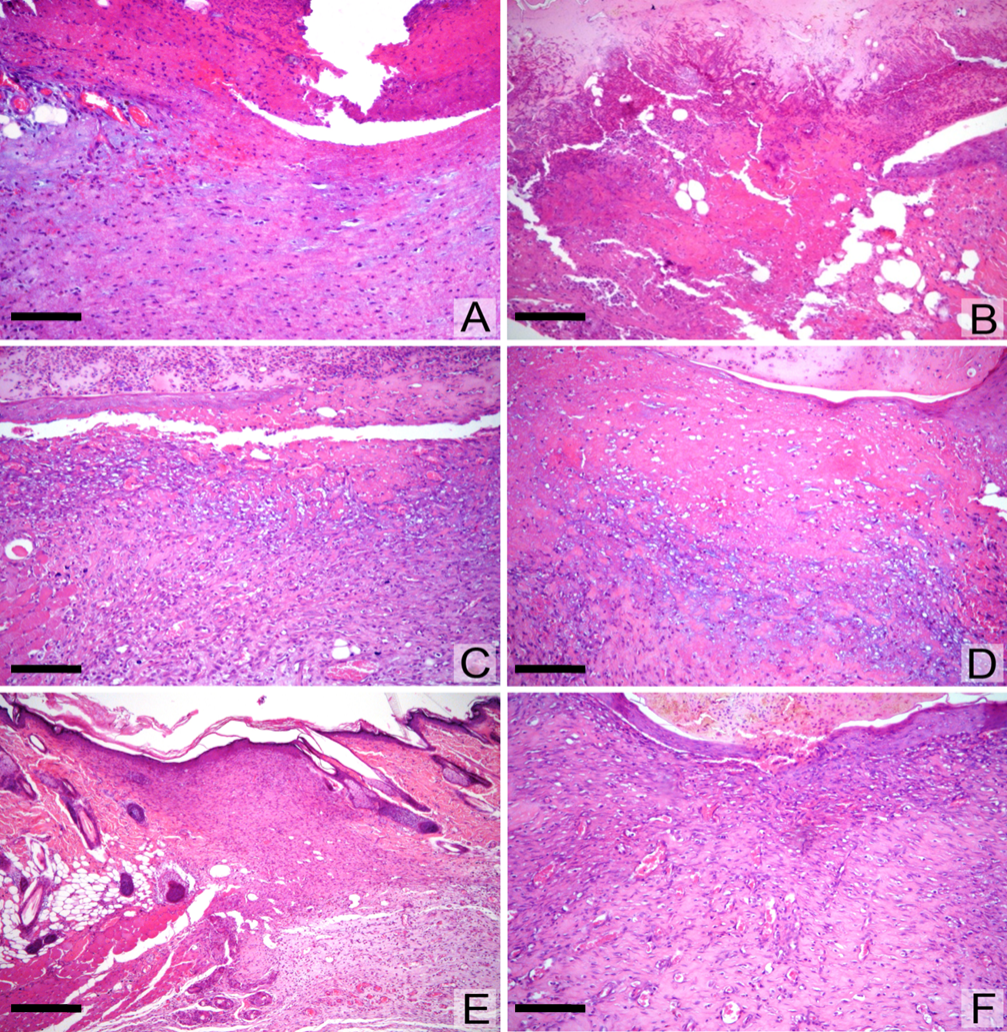
**Comparative histological analyses between the animals showing faster (A, C, E, F) and slower (B, D) healing.** Incipient cellular connective tissue on the 2nd day after injury (A); only necrotic-inflammatory material at the base of the ulcer in the other animal (B). Granulation tissue with collagen fibers deposited on the 4th day (C) and only slightly increased cellularity (D). Granulation tissue extensively collagenized on the tenth day, with almost complete re-epithelialization and discrete unevenness of the cicatricial area (E, F). [H&E, original magnification x120 (A-D, F), x50 (E). Bar = 110μm (A-D,F); 250 μm (E)].

Initially we analyzed the samples subjectively. On day 2, wound re-epithelialization had begun and higher cellularity and noticeable deposition of young collagen were observed in the animals treated with GCO and RCO. On day 4 (proliferation phase), the re-epithelialization was more pronounced in the GCO and RCO -treated animals. Cellularity was already lower in animals treated with GCO and RCO, and wound filling was greater in the control group animals. On day 10 (remodeling phase), the animals exhibited complete wound re-epithelialization. Fig 3 displays representative histological images on days 2, 4 and 10.

**Fig 4:**
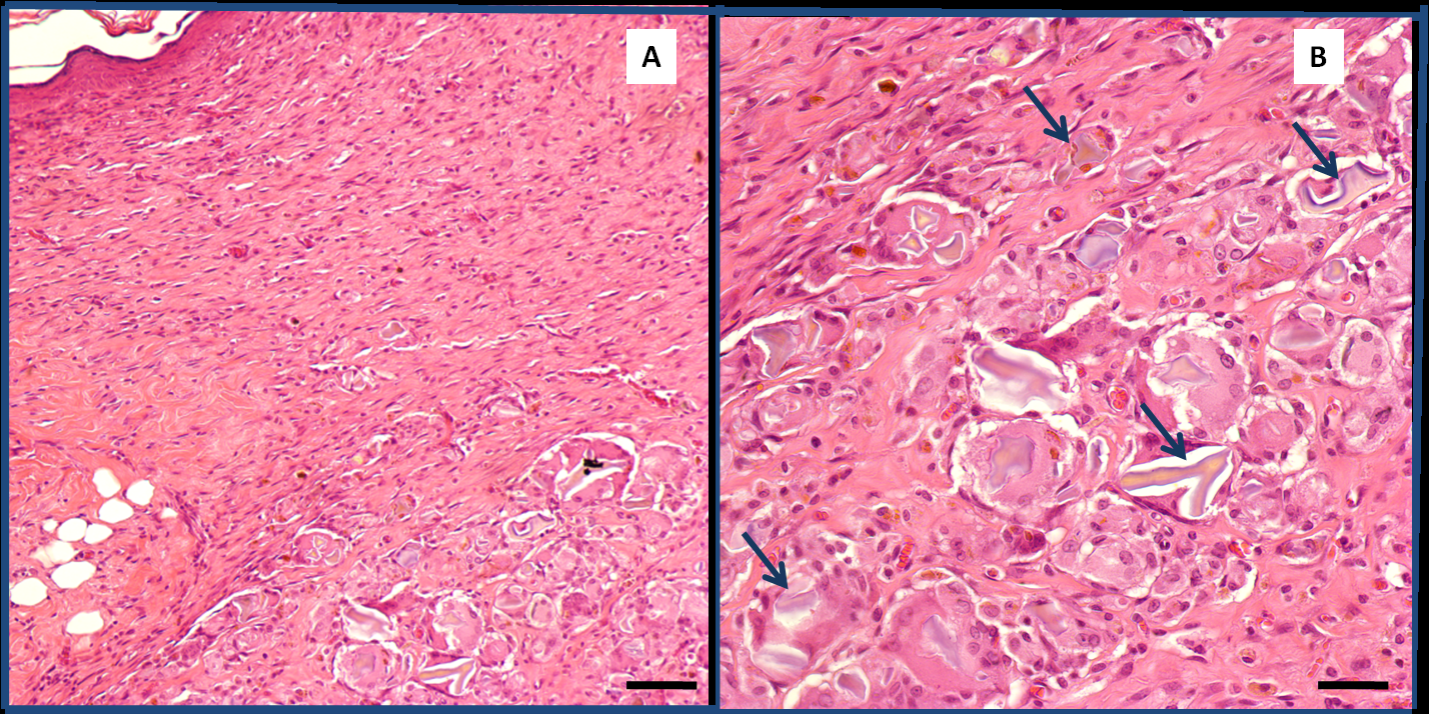
Representative image of wound treated with coffee ground oils. On the 10th day of coffee ground oil treatment, the inflammatory process persisted within the depth of the dermis under the scar. Several foreign body giant cells (arrows) contain various forms of blue-brown material. [H&E, original magnification x100 (A) and x400 (B)]. Bar = 15 μm (A) and 20 μm (B).

As previously stated, the animals treated with GCGO and RCGO showed worse wound healing (Fig 4) compared with the control animals, and they presented crusts and a persistent non-healed area.

**Fig 5:**
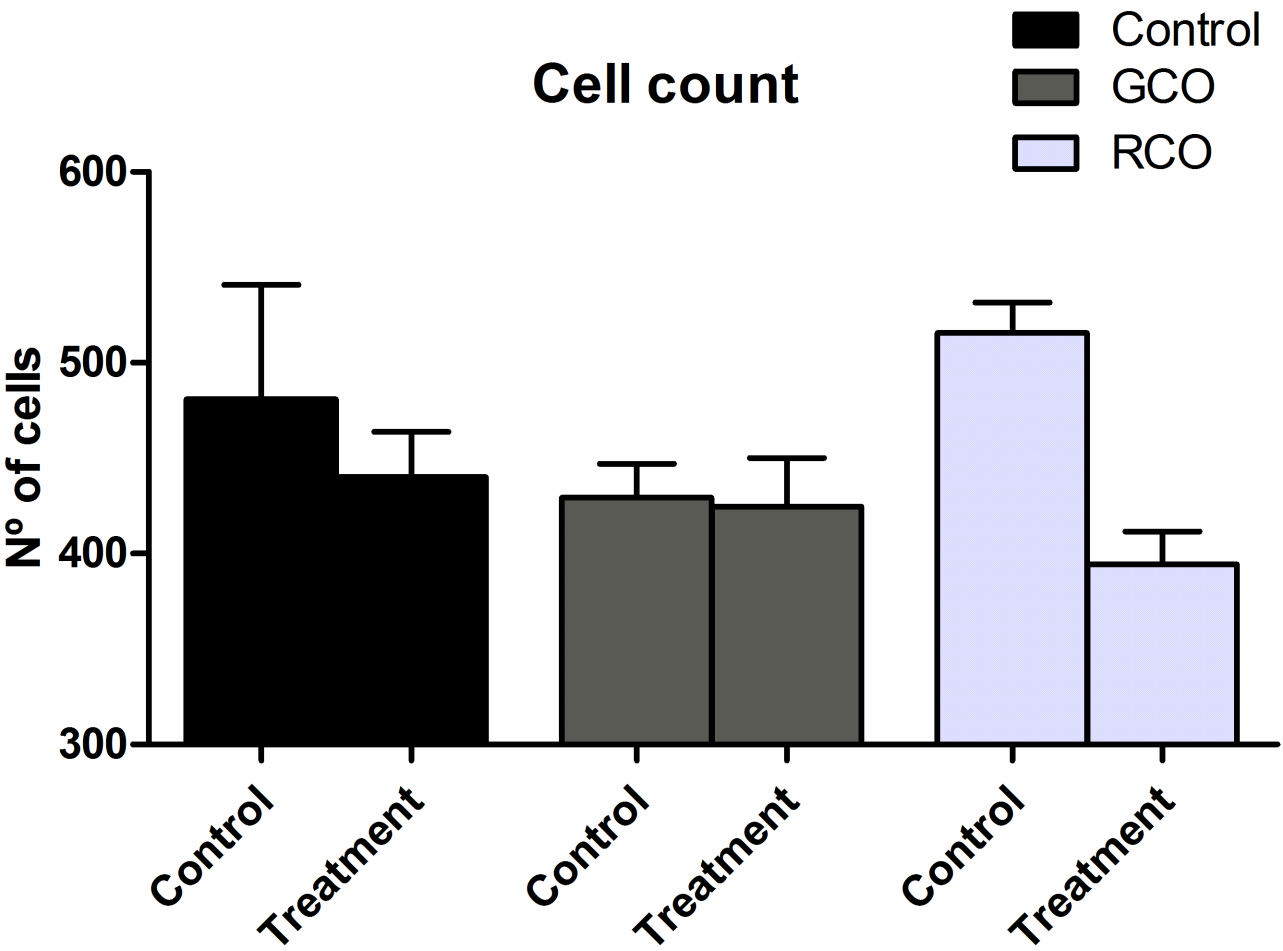
Wound site cellularity. Number of cells in the wound bed sites of the control and treatment sides of each group.

Fig 5 depicts the cell count results observed for the wounded dermis area of all groups at day 10 after surgery. General statistically significant differences were observed between the treatment sides (left is the control and right is the treatments, p = 0.001), although statistically significant differences were not observed among the treatments.

### SHG

**Fig 6:**
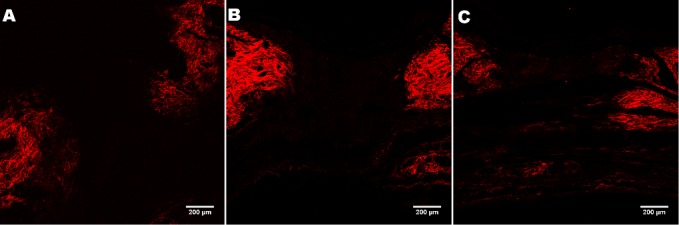
Representative images of wounds used in the SHG analysis. Image A (control group); B (GCO group); and C (RCO group). All images were taken with a 10x objective lens.

Statistically significant differences in collagen density were observed between the controls on the left side (4,404.83 ± 1,011.74 vs. 35,840.57 ± 8,940.17. p = 0.05). SS applied with MO (control group) presented lower values compared with MO applied with RCO, and the wounds treated with GCO had higher collagen densities than the control wounds (14,853.77 ± 9,034.13 vs. 70,089.94 ± 30,071.20, p = 0.05). The Fig 6 displays representative SHG images of wounds treated with MO, GCO and RCO.

### Real-time PCR

The real-time PCR results are presented as the fold change in expression relative to that of the control group (MO treatment).

**Fig 7:**
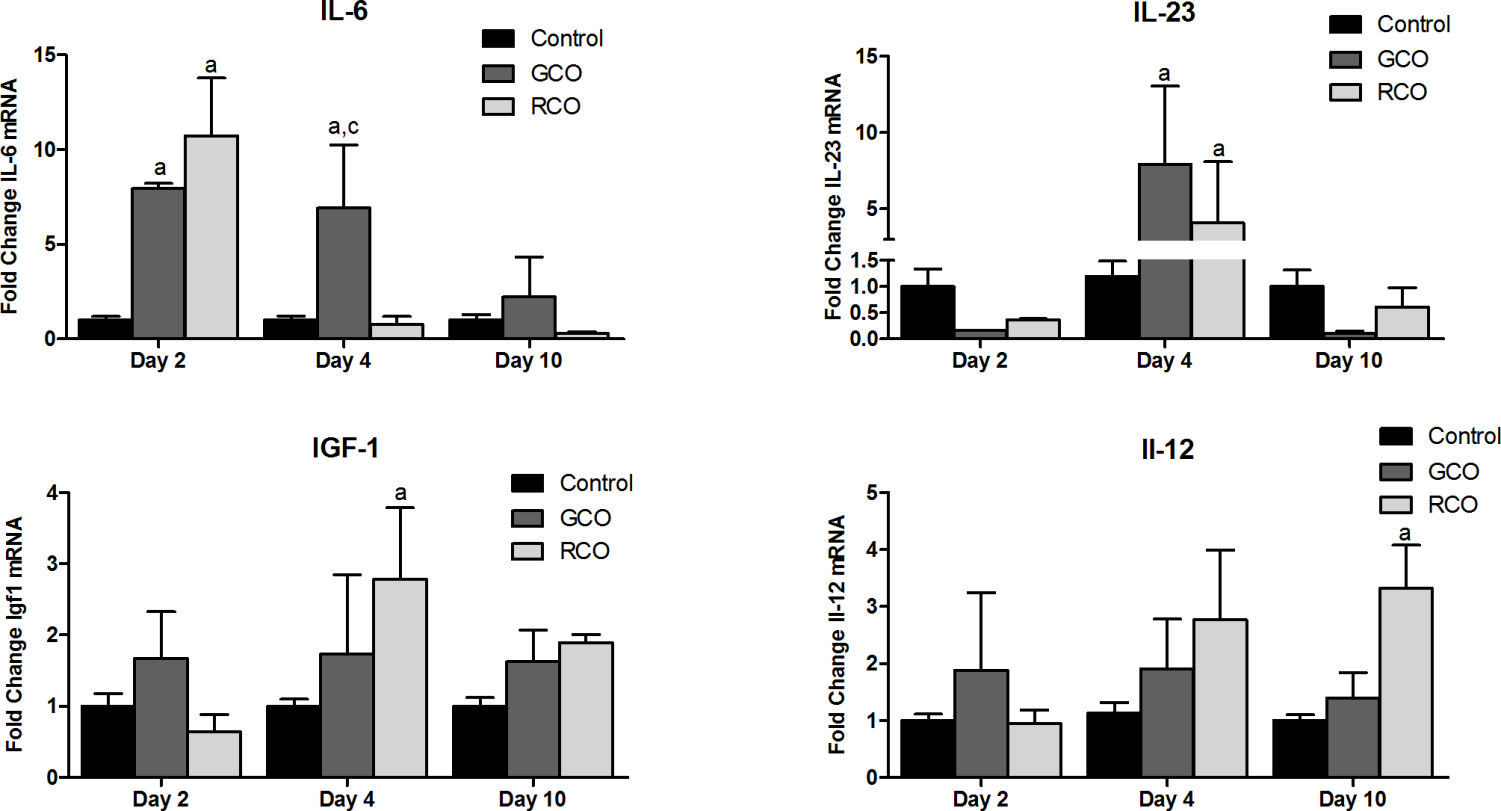
qPCR results for IGF-1, IL-6, IL-12 and IL-23. The letters above the bars represent a statistically significant difference between the group represented by the bar and the group represented by the letter as follows: a = control; b = GCO; c = RCO; d = GCGO; and e = RCGO. The p-values are as follows: IGF-1, p = 0.01; IL-12, p = 0.05; IL-6, p = 0.001 for RCO and p = 0.01 for GCO; IL-23, p = 0.001 for GCO, p = 0.05 for RCO and p = 0.05 for GCO vs. RCO.

We found that the GCO treatment increased the levels of IL-6 mRNA at the wound site throughout the experiment, especially on days 2 (7.94 ± 0.18-fold change vs. 1.00 ± 0.18 in the control) and 4 (6.90 ± 3.35-fold change vs. 1.00 ± 0.21 of the control group and 0.75 ± 0.44 of the RCO group) and increased the levels of IL-23 mRNA on day 4 (7.93 ± 5.13-fold change vs. 1.20 ± 0.30 of the control group). Animals treated with RCO had higher levels of IL-6 mRNA on day 2 (10.72 ± 3.05-fold change vs. 1.00 ± 0.18 in the control group), IL-23 on day 4 (4.10 ± 3.9-fold change vs. 1.20 ± 0.30 in the control group), IGF-1 on day 4 (2.78 ± 1.00-fold change vs. 1.00 ± 0.1 in the control group) and IL-12 on day 10 (3.32 ± 0.76-fold change vs. 1.00±0.10 in the control group). The other cytokines did not have statistically significant results. The results of the cytokine analysis of the tissue samples are shown in Fig 7.

The IGF-1, IL-6 and IL-23 (beneficial for wound healing, mainly in early stages) results are consistent with the improved healing observed in animals treated with RCO and GCO in this study. A positive IL-12 (anti-inflammatory, important at later stages) result was observed on day 10 in the RCO treatment.

### ELISA

ELISA tests were performed to assess whether the topical application of oils could induce a systemic response.

The animals treated with RCO and GCO had lower systemic levels of IFN-γ on day 4 compared with that the control animals (43.03 ± 5.79, 11.97 ± 11.63 and 196.45 ± 119.44 pg/mL, respectively), and the RCO treatment also reduced the levels of IL-4 on day 4 (0.9 ± 0.9 pg/mL vs. 13.36 ± 3.88 pg/mL) and adiponectin on day 10 (8,367.47 ± 1,302.80 pg/mL vs. 16,526.38 ± 1,858.37) compared with that of control. This treatment also increased the systemic levels of IFN-α throughout the entire experiment compared with that of the control (day 2, 52.53 ± 2.77 pg/mL vs. 21.20 ± 2.57; day 4, 46.98 ± 2.40 vs. 21.56 ± 1.74; day 10, 83.61 ± 15.08 vs. 25.69 ± 9.80).

**Fig 8:**
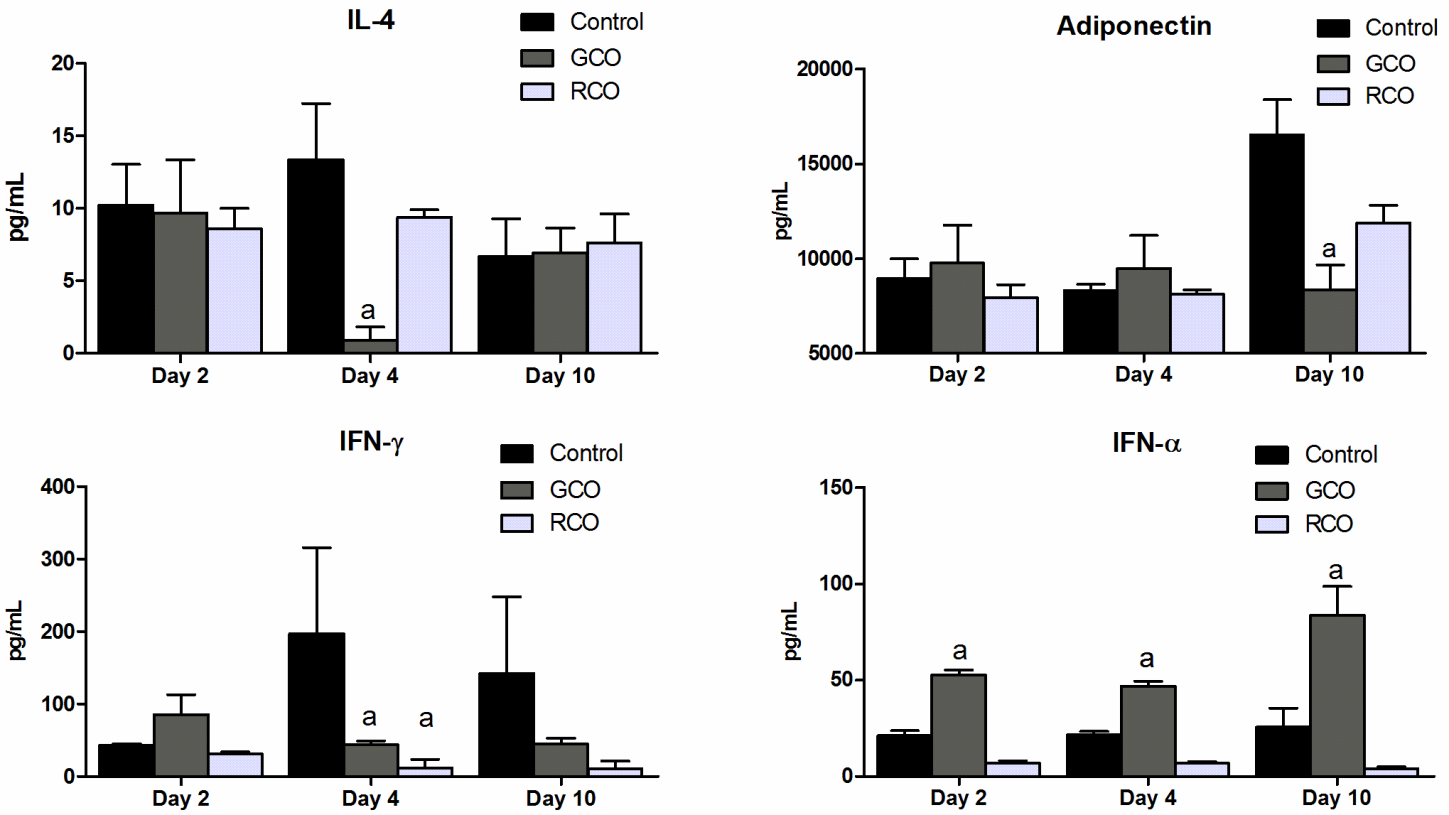
Graphs representing the ELISA results for IL-4, adiponectin, IFN-γ and IFN-α. The p-values are as follows: IL-4, p = 0.01; adiponectin, p = 0.001; IFN-γ, p = 0.01 for RCO and p = 0.05 for GCO and IFN-α, p = 0.05.

Fig 8 shows the cytokines levels that had a statistically significant response.

The circulating levels of IL-4 (beneficial for wound healing in initial stages) found in the GCO-treated animals are consistent with a better healing outcome. IFN-α, IFN-γ and adiponectin are cytokines important at later stages of healing and represent anti-inflammatory substances. The IFN-γ (for RCO and GCO) and adiponectin (for GCO) levels were consistent with the improved outcome found with these treatments. However, animals had higher systemic levels of IFN-α in the GCO treatment throughout the experiment.

### Coffee oil composition

The complete essential fatty acids compositions of the green and roasted coffee oils are detailed in Table 2. The fatty acids observed at high percentages were linoleic, palmitic, oleic and stearic acids in both oils. Statistically significant differences for the presence of these compounds were not observed.

**Table 2:** Essential fatty acid compositions of GCO and RCO.

Compound	% in GCO	% in RCO
C14:0	0.13	0.12
C15:0	0.04	0.03
C16:0	34.8	32.2
C16:1	0.04	0.04
C17:0	0.14	0.13
C18:0	7.79	8.45
C18:1	9.29	10.53
C18:2 trans	-	0.03
C18:2	41.86	42.4
C18:3	1.47	1.52
C20:0	2.99	3.06
C20:1	0.33	0.33
C22:0	0.84	0.87
C24:0	0.3	0.31

GCO, green coffee oil; RCO, roasted coffee oil.

Differences were observed in the phenolic composition of these oils. The main compounds of the GCO are listed in Table 3, and they consisted of hexadecanoic acid (trimethylsilyl ester), 9,12-octadecadienoic acid (methyl ester), 9,12-octadecadienoic acid (trimethylsilyl ester) and hexadecanoic acid. Additionally, 29.7% of the compounds were not identified.

**Table 3:** Phenolic composition of the GCO.

Compound Name	Molecular formula	Area (%)
Cyclohexanone, 2-methyl	C_7_ H_12_ O	0,09
1H-Pyrrole, 2,5-dihydro	C_4_ H_7_ N	0,27
Caffeine	C_8_ H_10_ N_4_ O_2_	1,46
Tetradecanoic acid, trimethylsilyl ester	C_17_ H_36_ O_2_ Si	0,13
Hexadecanoic acid	C_16_ H_32_ O_2_	6,6
Hexadecanoic acid, trimethylsilyl ester	C_19_ H_40_ O_2_ Si	29,54
9,12-Octadecadienoic acid, methyl ester	C_19_H_34_O_2_	15,62
9,12-Octadecadienoic acid, trimethylsilyl ester	C_21_H_40_O_2_Si	12,3
Octadecanoic acid, trimethylsilyl ester	C_21_H_44_O_2_Si	1,01
Docosanoic acid	C_22_ H_44_ O_2_	0,36
Eicosanoic acid, trimethylsilyl ester	C_23_ H_48_ O_2_ SI	1,36
Di-n-octyl phthalate	C_24_H_38_O_4_	0,94
9-Octadecenoic acid (Z)	C_18_ H_34_ O_2_	0,62
Unidentified compounds	-	29,7

The main compounds of the RCO as identified by GC-MS was hexadecanoic acid (trimethylsilyl ester), 9,12-octadecadienoic acid (methyl ester), hexadecanoic acid, octadecanoic acid and unidentified compounds (47,20%) as shown in Table 4.

**Table 4:** Phenolic composition of the RCO.

Compound Name	Molecular formula	Area (%)
2,4-Decadienal	C_10_H_16_O	0,12
1H-Pyrrole, 2,5-dihydro	C_4_H_7_N	0,04
Caffeine	C_8_H_10_N_4_O_2_	1,29
Tetradecanoic acid, trimethylsilyl ester	C_17_H_36_O_2_Si	0,1
Hexadecanoic acid, methyl ester	C_17_H_34_O_2_	0,04
Hexadecanoic acid	C_16_H_32_O_2_	9,08
Hexadecanoic acid, trimethylsilyl ester	C_19_H_40_O_2_Si	17,19
9,12-Octadecadienoic acid, methyl ester	C_19_H_34_O_2_	14,37
Octadecanoic acid	C_18_H_36_O_2_	8,81
Docosanoic acid	C_22_H_44_O_2_	1,06
α-Springene	C_20_H_32_	0,57
Tetratetracontane	C_44_H_90_	0,08
2,6,10,14-Tetramethylheptadecane	C_21_H_44_	0,05
Unidentified compounds	-	47,2

## Discussion

On day 2 (inflammation phase) the vessels were congested. A greater number of dilated vessels at this stage corresponded to faster healing because neutrophils and monocytes are attracted to the wound site [23]. On day 4 (proliferation phase), the vessels were no longer highly dilated, but their cells began to proliferate. Collagen was observed in greater quantities on day 4 for wounds showing earlier healing.

On day 10 (initial remodeling phase): Some animals still had remnants of young collagen (the same as the second day) that had not yet been replaced by the mature, thick fibers. A considerable area of the cavity formed in the experiment was already filled; however, the central area was still depressed relative to the adjacent normal skin [14].

The results summarized in Fig 5 shows that the group treated with RCO had a lower cell count, although the difference was not significant. This finding could be explained by the low number of animals used in each group.

As the wound healing process advances, fibroblasts should produce and deposit collagen into the extracellular matrix. SHG microscopy is a non-linear imaging technique that provides morphological information on biological tissues. Collagen fibers produce a good SHG signal [24]. SHG has been used for the study of the distribution, orientation and quantification of collagen in different tissues, diseases such as tumors, scars and for the evaluation of skin photodamage. In the RCO-group, the lesions treated with MO, had a higher collagen density compared with the control group; and in the GCO-group, the lesions treated with MO presented a similar result. This finding might suggest a systemic effect of roasted coffee oil when topically applied to animal skin.

As previously stated, the wound healing response is regulated by a large quantity of cytokines secreted by the associated cells [25]. Of the cytokines analyzed in our study, IGF-1, IL-4, IL-6, IL-23 are pro-inflammatory substances that are important at the initial stages of wound healing, whereas adiponectin, IL-12, IFN-α and IFN-γ are anti-inflammatory substances that are important at later stages of wound healing.

Adiponectin is secreted from adipocytes and is a metabolic regulator of lipids and glucose [26];. A study using a mouse model found that when adiponectin is directly injected into the wound edge, accelerated wound healing occurred [27]. Our results showed that the use of GCO decreases the level of circulating adiponectin compared with the control group. In another study on adiponectin’s role in wound healing, the authors found that it suppressed keratinocyte differentiation and proliferation and induced apoptosis [28]. The use of RCO and GCO reduced the serum adiponectin levels during all time points of our study, which is consistent with the results found in the histological evaluation and the results of the study performed by Kawai and colleagues. However, further studies on the action of adiponectin in skin wound healing are necessary to clarify its role. We found that treatment with GCO can increase the levels of IFN-α produced by the animals on days 2, 4 and 10 because it was detected in serological analysis by ELISA throughout the treatment period. IFN-α plays a key role in innate immune responses and primes the immune system for subsequent adaptive immune responses. Plasmacytoid dendritic cells (pDCs) are the major source of IFN-α [29] and represent a rare population of circulating cells that specialize in the production of large amounts of type I IFNs [30]. pDCs have recently been identified as a major IFN-α inducer in wounded mouse and human skins, and these cells may be able to distinguish self-DNA from skin cells injured at the wound site, which lead to increased IFN mRNA expression via the Toll-like Receptor (TLR)-7 and TLR9 endosomal pathways and skin reepithelialization [30].

The use of GCO and RCO decreased the systemic level of IFN-γ on day 4 compared with the other treatments. IFN-γ exerts regulatory functions to limit the tissue damage associated with inflammation [29]. A study performed by Tanno and collaborators found that skin wound healing in rats was enhanced by inducing the early-phase production of IFN-γ. Into that group, the IFN-γ roles may have differed between the early (activate macrophage phagocytosis and promote VEGF production) and late stages (inhibit TGF-β) [31].

IGF-1 is a growth factor produced by fibroblasts and other epithelial cells, and it plays an important role in re-epithelialization and granulation tissue formation during the wound healing process [32]. One of its roles is vascular endothelial insulin/IGF-1 signaling in the homeostasis of the skin vasculature and in neovascularization on the skin during wound healing [33]. Because of the role of IGF-1 in angiogenesis, promoting high levels in the early stages of the wound healing process is important. In this study, we found higher levels of IGF-1 mRNA expressed in the skin of the animals treated with RCO on day 4 of treatment compared with that of the control animals.

IL-6 plays an important role at the beginning of the inflammatory response and act as a chemotactic factor for leucocyte recruitment. Butzelaar and colleagues found that lower levels of this cytokine in the early stage of skin wound healing could lead to hypertrophic scar formation [34]. Our group found higher levels of IL-6 mRNA expressed at the wound site in the animals treated with GCO on days 2 and 4 and with RCO on day 2, and the RCO-treated animals showed better wound healing among all groups of this experiment. None of the animals in our study had hypertrophic scars, which is consistent with the results found by other researchers. Another study stated that IL-6 deficiency can lead to hyper-inflammatory macrophage activation, which could produce a chronic wound state because of the destruction of the newly formed tissue [35].

IL-12 is a cytokine primarily produced by monocytes, macrophages and dendritic cells and is likely involved in the development of cell-mediated immunity [36]. This cytokine induces IFN-γ synthesis [37], and a previous study demonstrated that the administration of additional IL-12 on acute skin wound sites increased the local metabolic rate, which benefited the healing process [38]. The results of this study suggest that the topical use of RCO in wounds may have promoted the local synthesis of IL-12 without interfering with its systemic levels.

The relatively recently discovered IL-23 cytokine is involved in the pathogenesis of psoriasis (an injection of this cytokine can lead to psoriatic lesions formation on the skin), and it is related to the IL-12 and Th17 responses [39]. Our study found higher levels of IL-23 mRNA in GCO and RCO-treated animals on day 4. One of the possible actions of this cytokine is the hyperproliferation of keratinocytes (a sign of psoriasis). This result is consistent with the IL-12 data and may indicate possible crosstalk among these substances in skin cell proliferation [40].

The differences observed between the GCO and RCO-treated animals could be associated with the phenolic composition of these oils, because statistically significant differences in the fatty acid composition were not observed. Our results showed higher quantities of hexadecanoic acid and trimethylsilyl ester in the GCO and higher quantities of octadecanoic acid in RCO. The percentage of unidentified compounds (29,7% in GCO and 47,20% in RCO) was an interesting finding. Several studies have focused on the efficacy of using coffee oil for improving skin, and green beans have generally been used as the source [10, 13]. Further studies are needed to detail the total composition of these oils.

## Conclusions

In summary, the results presented here encourage the use of RCO on skin wounds. Both coffee oils (GCO and RCO) produced systemic effects as observed in the serum cytokine levels and SHG analysis results. Further studies may identify the most active molecules and the mechanisms of action.
